# Evaporation of a sessile water drop and a drop of aqueous salt solution

**DOI:** 10.1038/s41598-017-15175-1

**Published:** 2017-11-07

**Authors:** S. Y. Misyura

**Affiliations:** 1 Kutateladze Institute of Thermophysics of the Siberian Branch of the Russian Academy of Sciences, Lavrentiev Ave. 1, Novosibirsk, 630090 Russia; 2 National Research Tomsk Polytechnic University, 30 Lenin Ave, Tomsk, 634050 Russia

## Abstract

The influence of various factors on the evaporation of drops of water and aqueous salt solution has been experimentally studied. Typically, in the studies of drop evaporation, only the diffusive vapor transfer, radiation and the molecular heat conduction are taken into account. However, vapor-gas convection plays an important role at droplet evaporation. In the absence of droplet boiling, the influence of gas convection turns out to be the prevailing factor. At nucleate boiling, a prevailing role is played by bubbles generation and vapor jet discharge at a bubble collapse. The gas convection behavior for water and aqueous salt solution is substantially different. With a growth of salt concentration over time, the influence of the convective component first increases, reaches an extremum and then significantly decreases. At nucleate boiling in a salt solution it is incorrect to simulate the droplet evaporation and the heat transfer in quasi-stationary approximation. The evaporation at nucleate boiling in a liquid drop is divided into several characteristic time intervals. Each of these intervals is characterized by a noticeable change in both the evaporation rate and the convection role.

## Introduction

The evaporation of droplet aqueous solutions is widely used in power apparatus and chemical technologies, and is often observed in medicine and biology. Predicting the droplet evaporation rate of these solutions is necessary for the development of such important technologies as: inkjet printing, coating technology, medical diagnostics and microelectronics cooling. Correct modeling of the evaporation of aqueous solutions is important to increase the efficiency of heat pumps. Vapor absorption is used in absorbers, and vapor evaporation is carried out in desorbers of lithium-bromide heat pumps. Both in the absorber and the desorber, the liquid film breaks, and dry spots, jets and droplets are formed on the heat exchanger surface. Typically, to consider droplets evaporation, the stationary modeling of quasi-isothermal behavior is used. In this case, physical coefficients of an aqueous solution weakly change over time, and boundary conditions are approximately constant. In reality, we usually face nonstationary and nonisothermal conditions. However, there are a large number of key components that are interrelated and hardly separable. It is difficult to distinguish the influence of each individual factor on evaporation and heat mass transfer. Therefore, to date, the important question remains: what key factors are predominant in the study of complex solutions at droplets evaporation? The most advanced is modeling for droplets with a small droplet diameter *d* (*d* < 1–2 mm) at low heat fluxes. Typically, for a small sessile droplet, only gas diffusion is considered. At that, liquid convection and Marangoni force are neglected^1,2^. The droplet evaporation without nucleate boiling was studied in refs^1–5^. The heat transfer in the droplet is strongly dependent on the wall material. Wall materials with high thermal resistance lead to a significant decrease in the evaporation rate^6,7^. When changing the wall properties, the evaporation character may change. In this case, the transition from Leidenfrost regime to nucleate boiling mode is possible^8^. At bubbles nucleation into a sessile droplet and high heat flux the evaporation character becomes more complex. The droplet wetted diameter, the evaporation rate, and the wall and interface temperature change in time. The droplet equilibrium is broken, and we face a sliding contact line of the sessile droplet. In this nonstationary case, there are several characteristic time intervals at droplet evaporation^9^. There are many factors that influence the nucleate boiling. The nucleate boiling behavior is determined by RMS^10,11^ and wall surface topology (fractal dimension)^12^. The structured surface may both increase and decrease the wettability and the heat transfer. The evaporation behaviors of the sessile droplet on the structured and the smooth wall are markedly different^13,14^.

The deposition behavior of nano particles in colloidal solutions depends on the evaporation rate, and deposition pattern is of interest and helps diagnosing the solutions. Particle depositions into a droplet solution are investigated in refs^15,16^. The behavior of a multicomponent solution noticeably differs from that of a one-component liquid. At evaporation of aqueous salt solutions only water evaporates (desorbs), and salt remains in the solution. For aqueous salt solution, the equilibrium vapor pressure and the interface temperature are determined by salt concentration. Systematic studies of properties of aqueous salt solution are presented in refs^17,18^. One of the effective ways for increasing the heat pump efficiency is to increase the wall overheating and the heat transfer coefficient *α*. In the transition from evaporation without nucleate boiling to boiling with vapor bubbles, the coefficient *α* grows drastically. A bubble at pool boiling grows due to liquid evaporation from the entire bubble interface^19^, and the bubble growth on a wall occurs mainly due to evaporation of a liquid microlayer on the wall^20^. At nucleate boiling of aqueous salt solution the bubble growth rate decreases significantly in comparison with boiling in pure water. With bubble growth in an aqueous solution, the bubble contact line is unstable, and a dry spot under the bubble does not form^21^. The fact of dry spot absence is very important, and this allows substantially raising the critical heat fluxes. At nucleate boiling, properties of solutions rapidly change for a short time interval that also complicates simulation of evaporation and heat transfer. Thermodynamic properties of salt solutions in a wide range of physical parameters are presented in refs^17,18,22,23^.

One of the important problems for technical devices using high temperature evaporation is the formation of crystalline salt hydrates. So, at high-temperature flow of the salt solution in a minichannel, there may arise the emulsion flow regime with the formation of the crystalline hydrate film on the channel walls, which leads to channel clogging and to flow breakup^24^. The growth of salt hydrates and their morphology are considered in ref.^25^. Qualitative behavior of the drop evaporation rate in saturated and supersaturated solutions differs markedly. This difference is due to the dependence of the no-slip condition of the contact line on the degree of supersaturation and on the place in a drop where the first crystals of salt (crystallohydrates of salt) are formed^26^. The presence of cationic surfactant CTAB and nonionic surfactant Tween 80 in an aqueous solution of NaCl salt delays the crystallization of sodium chloride with evaporation^27^. On the free drop surface, crystals of NaCl salt form and move in the direction from the contact line to the center of the drop^28^. This direction of motion is realized due to capillary forces^29^. Peculiarities of nonisothermal evaporation of droplets of lithium bromide–water solution are considered in refs^30,31^.

When modeling the evaporation and the non-isothermal absorption, the convection effect is usually neglected. It is supposed that the entire phase transition heat is spent for conductive thermal conductivity of liquid and for radiation^32–37^, which is not always correct, even at low mass and heat fluxes and with little change in water concentration of the aqueous solution. The degree *a* in the law of drop evaporation (the radius *R* continuously decreases with time) *R*~(*t*_0_-*t*)^*a*^ depends on the molecular weight of liquid^38^. To realize the vapor buoyancy, the vapor density must be significantly different from the density of air. For a solution (water-ethanol) of a droplet with small radius (less than 1 mm, the droplet evaporates without heating with *T*_*w*_ ≈ 25 °C) it is necessary to consider the thermal effect of the heat of evaporation, and the influence of gas convection and Marangoni forces are negligible^39^. An influence of solutally driven natural convection on the evaporation rate was considered in ref.^40^. The convection has to be taken into account only if *Ra* > *Ra*_*cr*_ = 1000–2000 (*Ra*_*cr*_ is the critical Rayleigh number)^40^. The combined effect of the natural convection and the nucleate boiling is considered in ref.^41^. In the last decades, the attention of researchers to the simulation of evaporation, taking into account the combined effect of natural convection and gas diffusivity, has increased. It is determined that in addition to the diffusion vapor flux and Stefan flow, it is necessary to take into account the effect of the natural gas convection on the droplet evaporation rate^42^. An increase in a droplet radius results in a multiple increase in the evaporation rate of liquids with low latent vaporization heat^43^. Even at evaporation of a water drop with large radius *r* without heating a wall, there is a significant effect of natural convection on the droplet evaporation law^44^. In the presence of a transverse gas flow from a permeable wall, the similarity of the laws of heat transfer, friction and mass transfer *St* = *C*_*f*_/2 = *St*_*d*_ may be significantly violated (*St* is the Stanton number, *St*_*d*_ is the diffusion Stanton number, and *C*_*f*_/2 is the dimensionless friction coefficient)^45^. The analogous situation is observed in the problems of a permeable plate and foreign gas blowing into the boundary gas layer. The vapor density *ρ*_*s*_ (equilibrium partial vapor density at the drop interface) may be considered as an initial foreign gas density. With increasing blowing intensity in the form of a parameter ${\bar{j}}_{w}$ (*Re*)^0.5^ (where ${\bar{j}}_{w}$ = *j*_*w*_/*ρ*_0_ *u*_0_ is the relative blowing rate, and *j*_*w*_ is the mass diffusion flux), the shear stress (*C*_*f*_/2) decreases continuously, but heat and mass fluxes (*St* and *St*_*d*_) can both increase and decrease and have extremums^45^.

Experimental investigation of mixtures and foreign inclusions in water droplets under high-temperature gas was considered in ref.^46^. Evaporation, boiling and explosive breakup of heterogeneous droplet in a high-temperature gas was presented in refs^47,48^.

Thus, the joint influence of several key parameters (vapor-gas diffusion, the Stefan flow and free convection in liquids and in gas) at non-isothermal evaporation has not been sufficiently studied, especially at a high heat flux. When simulating the non-isothermal evaporation and the heat and mass transfer it is important to know what factors are crucial and which can be neglected. In problems associated with droplet evaporation of an aqueous salt solution, the character of non-isothermal evaporation fundamentally differs from single-component liquids. Studying the influence of components’ concentrations on the qualitative and quantitative behavior of droplet evaporation is also an important scientific task.

## Droplet evaporation without nucleate boiling

**Figure 1:**
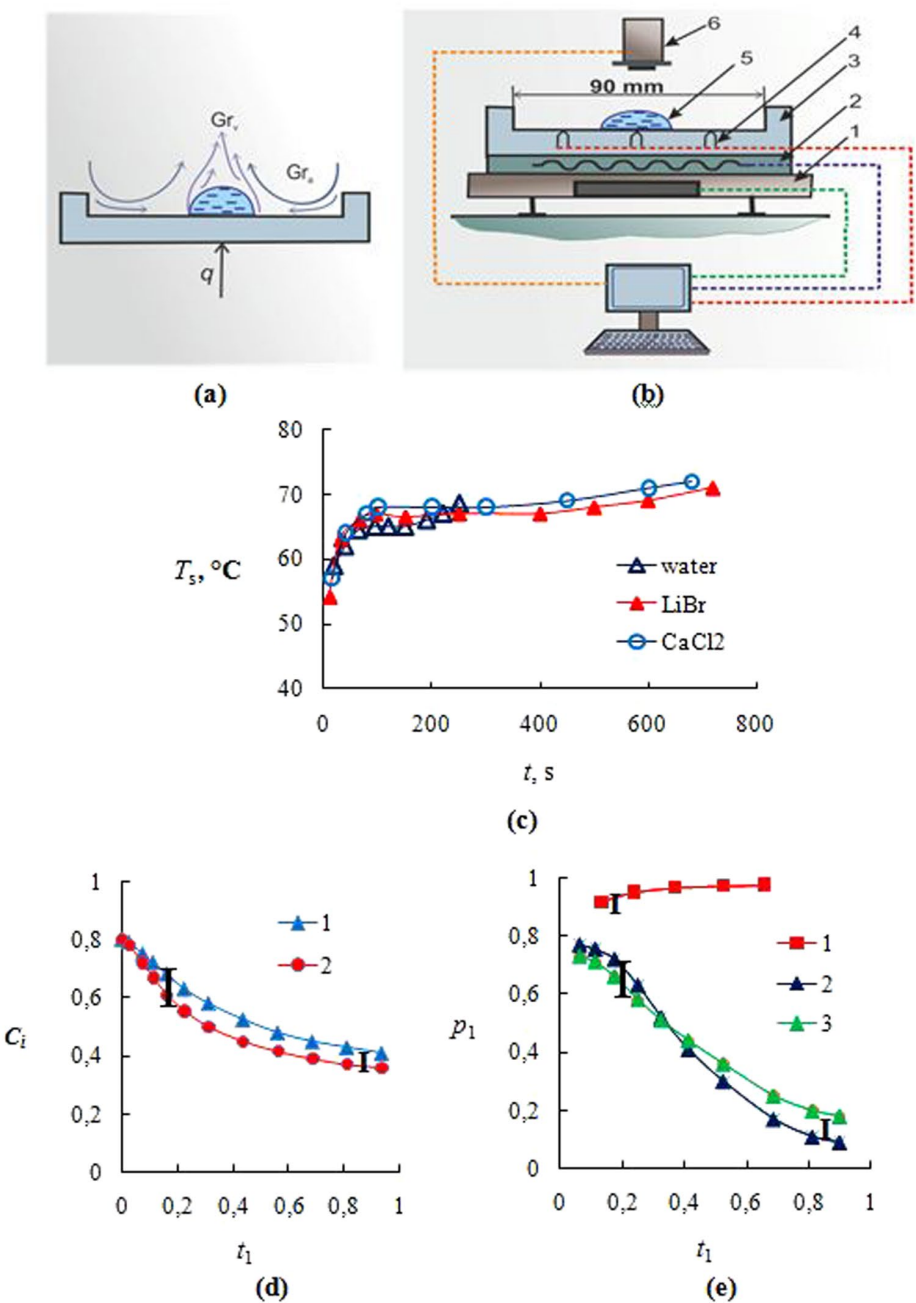
(**a**) Free convection over a drop (*Gr*_v_) and above the heated wall (*Gr*_a_); (**b**) The scheme of experimental setup: 1 – electronic balance; 2 – heater; 3 – metal working section; 4 – thermocouple; 5 – droplet; 6 – thermal imager; (**c**) Temperature (*T*_s_) of liquid-gas droplet surface (*V*_0_ = 250 μl; *T*_w_ = 75 °C); (**d**) The mass concentration of water (*C*_0_) for the drop of aqueous salt solutions of CaCl_2_ (curve 1) and LiBr (curve 2): *C*_0_ = 0.8; *V*_0_ = 250 μl; *T*_w_ = 75 °C; (**e**) The equilibrium partial vapor pressure for the water drop (curve 1); for the aqueous salt solution of LiBr (curve 2) and for the aqueous salt solution of CaCl_2_ (curve 3): *C*_01_ = 0.2 (*C*_01_ is the initial mass salt concentration); *V*_0_ = 250 μl; *T*_w_ = 75 °C: (*t*_1_ = *t*/*t*_cr_ (for the aqueous salt solution, *t*_cr_ is the start time of crystallization); *t*_1_ = *t*/*t*_t_ (for the water drop, *t*_t_ is the total evaporation time); *p*_1_ = *p*_s_/*p*_max_ (*p*_max_ is the maximum partial vapor pressure for the water drop (*t* = *t*_t_)).

A schematic layout of the experimental setup is presented in Fig. 1(a): 1 – electronic balance; 2 – heater; 3 – metal working section; 4 – thermocouple; 5 – liquid (water or aqueous salt solutions LiBr, CaC_2_); and 6 – thermal imager. The experiments were carried out at relative air humidity of 40%, ambient air temperature of 21 °С and ambient air pressure of 1 bar. The initial temperature of drops was equal to the ambient air temperature of 21 °С. The sessile drops were placed on the horizontal heated wall of the working section. The working section was titanium square section (*a* × *b* × *h*, where *a* = *b* = 90 mm, and the height *h* = 50 mm). Drops were placed on the heated wall by a microdoser, located perpendicular to the wall surface. The drop radius *r*_0_ was calculated as an average value for the entire area of the wetted drop surface. The difference in the drop radius for repeated experiments did not exceed 6%. The thermocouples for wall temperature measuring were located near the wall surface (0.2–04 and 2 mm from the surface). The wall temperature was kept constant in an automatic regime with the accuracy within ±0.5 °С. The drop interface temperatures were determined with the help of the thermal imager NEC R500 (resolution of infrared camera was 640 × 512 pixels). The error of thermal imaging measurements associated with a change in salt concentration from 10% to 60–65% was 1–3%. Thus, the change in salt concentration did not affect temperature measurements. The experimental setup was located under a shell that provided constant ambient humidity and temperature. Before each experiment, distillate was degassed by means of boiling, to reduce the amount of dissolved gas. Degassed distillate was used in all experiments. The values of drop areas have showed good reproducibility in repeated experiments. At water evaporation the mass of the solution decreased. The current mass value *m*_i_ was measured automatically by electronic scales (Fig. 1(a)). The current value of the drop volume was determined as *V*_i_ = *m*_i_/*ρ*_i_, where *ρ*_i_ is the current density of the solution, determined according to the average temperature of the solution and the average concentration in the droplet. The value of initial mass salt concentrations of aqueous salt solutions of LiBr and CaCl_2_ (*С*_01_) was determined using densimeters. Current salt concentrations of aqueous salt solutions *С*_*i*_ were determined by a weight method. The setup was placed on the precision balance. Since the salt mass does not change with time, it is easy to determine the current concentration values. As the salt concentration grows with time, the water concentration, on the contrary, decreases over time (*C*_0_ = ${С}_{{H}_{2}O}=1-{С}_{CaC{l}_{2}}$; where ${C}_{{H}_{2}O}={m}_{{H}_{2}O}/{m}_{mix}$; *C*_01_ = ${C}_{CaC{l}_{2}}={m}_{CaC{l}_{2}}/{m}_{mix}$; ${m}_{{H}_{2}O}$, ${m}_{CaC{l}_{2}}$, ${m}_{mix}$ is the mass of water, salt and solution in the drop). The increase in salt concentration leads to a change in the equilibrium partial vapor pressure *p*_*s*_ at the interface as well. At the known equilibrium values of temperature and concentration *C*_*s*_, the vapor density *p*_*s*_ is uniquely determined by equilibrium curves. The maximum error in mass measurements was 10–15% for large times at low evaporation rate. The gravimetric method allowed determining only the average mass concentration for the whole drop but not a local concentration for a transverse concentration profile. The systematic error was due to the fact that the equilibrium drop concentration (*C*_s_) differed from the average concentration by no more than 10%. The low evaporation rate resulted in a low transverse gradient of salt concentration near the drop interface. The relative measurement error of the equilibrium pressure (*p*_s_) did not exceed 20%. In all experiments with no nucleate boiling for both water and salt solution, the radius of the droplet was constant for most evaporation time *R*_0_ = *R*_*i*_ = const. The radius of the droplet was recorded by video camera, located above the surface of the drop. Figure 1(a) schematically shows two types of free gas convection, realized on the surface of the horizontal heater: free convection of air over the heated wall (the length of the work area significantly exceeded the droplet diameter), and free convection of vapor over the drop surface. The temperature at the droplet interface was slightly lower than the wall temperature without a drop due to evaporative cooling. Over the drop during the whole time of evaporation, the temperature difference Δ*T* = *T*_s_ − *T*_w_ (*T*_w_ is the wall temperature, *T*_s_ is the temperature of the gas-liquid surface) between the free surface of the drop and the wall changed. The graph of temperature changes at the droplet interface is shown in Fig. 1(b). The temperature *T*_s_ of liquid increased for the initial time due to the drop heating. A drop of ambient temperature (21 °C) was placed on a heated wall. After the water drop heating, the temperature *T*_s_ was quasi-constant and increased when the drop height due to evaporation substantially decreased from 4–5 mm to 0.5–1 mm. The temperature *T*_s_ of the aqueous solution of salt increased, because over time, the salt concentration in the droplet increased, and the evaporation rate *j* decreased. This reduction was conditioned by a decrease in the equilibrium partial vapor pressure above the gas-liquid interface. The Grashof number for air (thermo-gravitational air flow over the heater) is *Gr*_a_ = 5·10^5^, and for free convective vapor flow over a drop *Gr*_v_ = 1·10^4^. The critical *Gr*_cr_, at which the gas loses its stability, and the motion starts, is about 1000–2000. Thus, the numbers of *Gr* in these experiments significantly exceed the critical values that proves the intensive free convection. It should be noted that the attempt to evaluate the role of free convection in the increase of *j* through the equation of heat transfer and through the heat transfer coefficient is incorrect. Intensification of heat exchange by free convection is estimated at 10–20%. And intensification of evaporation flow is much higher because of a violation of the analogy between heat transfer and mass transfer^45^.

The evaporation rate of a small water droplet in time is quasi-constant^49^. Quite different is the behavior for a salt solution droplet. Over the time of evaporation, the salt concentration in the droplet grows, since only water transforms into vapor, and salt remains in the aqueous solution.

Figure 1(a) presents experimental data of the mass concentration of water for a sessile drop of aqueous salt solution (LiBr and CaCl_2_). The initial concentration of water was 0.8. The initial drop volume was 250 µl. The wall temperature *T*_w_ = 75 °C, which was maintained constant with an automatic control.

Figure 1(b) demonstrates the curves of the equilibrium partial vapor pressure for a water drop (curve 1) and for aqueous salt solutions of CaCl_2_ and LiBr (curves 3 and 2). The equilibrium vapor pressure *p*_s_ for water is quasi-constant; and for aqueous salt solutions *p*_*s*_ significantly decreases with time, and it is reduced almost ten times when the salt solution approaches the crystallization point. At the end of the evaporation time, values of *p*_*s*_ are quasi-constant.

The evaporation rate will decrease with time due to the tendency of the solution to equilibrium, as the driving force of desorption Δ*C* is close to null (Δ*C* = *C*_*i*_ − *C*_*s*_, where *C*_*i*_ is the average concentration of water in the droplet, and *C*_*s*_ is the equilibrium concentration of water on the interfacial surface). A decrease in the density of mass flow of water over time can be clearly seen from expression (1)^36^

1 $$j=-\rho D{(\frac{\partial C}{\partial y})}_{y=0}=\frac{\rho \sqrt{D}({C}_{0}-{C}_{so})}{\sqrt{\pi t}},$$

where *ρ* and *D* are the density and the coefficient of solution diffusion, *t* is the time, *C*_0_ is the initial water concentration, *C*_s0_ is the water equilibrium concentration for droplet free surface and for *T* = *T*_0_ (*T*_0_ is initial solution temperature), *j* (kg/(m^2^s), *C* = *ρ*_*water*_/*ρ*_*mix*._, and *ρ* = *ρ*_*mix*_ is the density of mixture.

Expression (1) is derived for the simplified case of absorption, when *T*_*s*_ is constant. This condition occurs when the rate of absorption or evaporation is very low, and the heat of absorption (desorption) may be neglected. In addition, we consider either the case of an infinitely thick layer or small times, when the diffusion layer has not reached the bottom of the liquid layer. In addition, the expression (1) is obtained in the absence of convection both in the liquid and in the gas phase, and in the absence of heat release on the solid wall; therefore it is not used below, but helps to qualitatively evaluate the role of diffusion in liquids and the role of Δ*С*.

**Figure 2:**
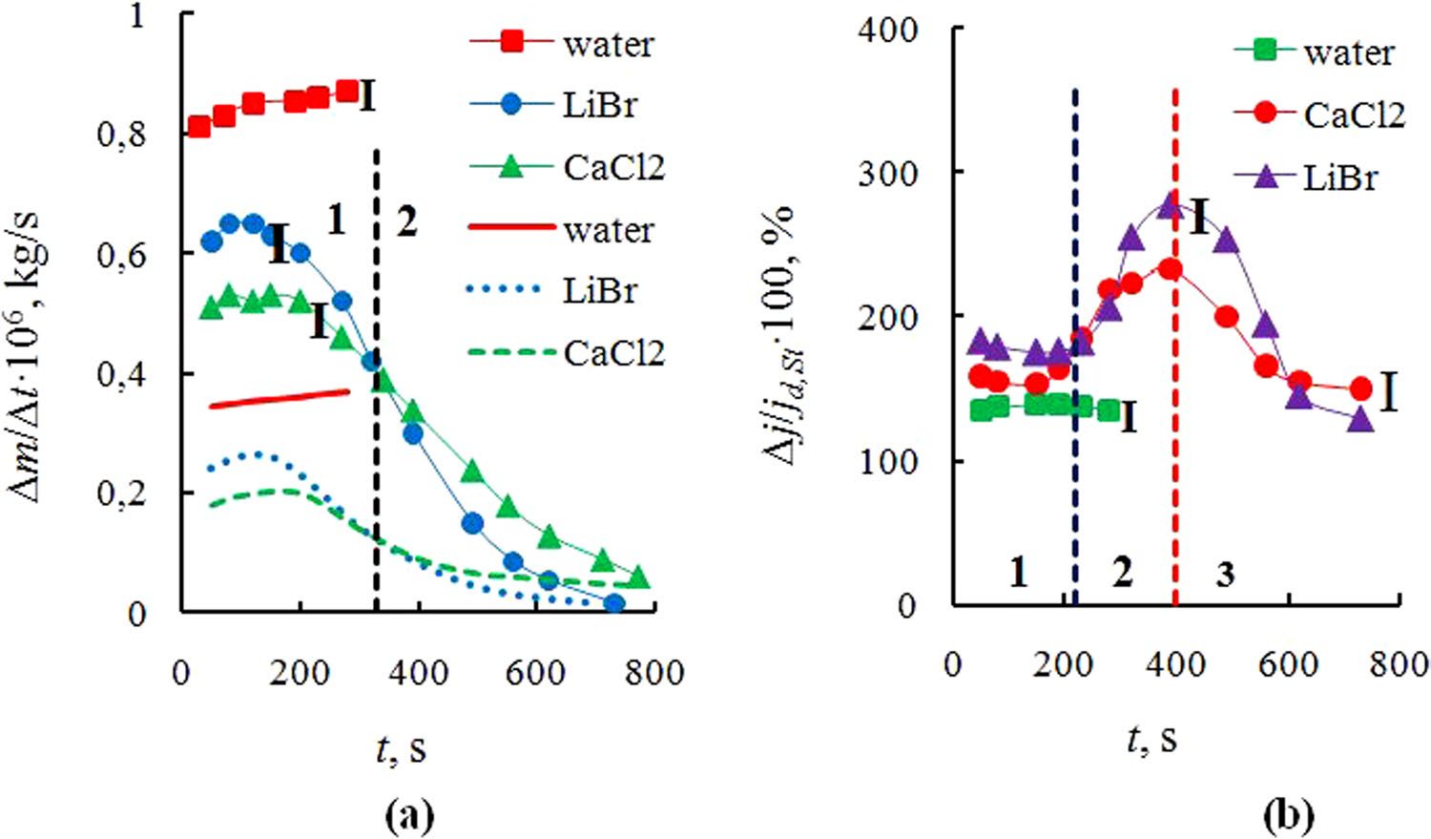
(**a**) The evaporation rate Δ*m*/Δ*t* with time *t* (*C*_01_ = 0.2 (the initial mass concentration of salt); *V*_0_ = 250 μl; *T*_w_ = 75 °C): 1–3 – experiment; 4–6 – modeling; curves 1, 4 – for water drop; curves 2, 5 – for aqueous salt solution of LiBr; and curves 3, 6 – for salt solution of CaCl_2_; (**b**) The ratio of natural convection and gas diffusion at evaporation, Δ_1_ = Δ*j*/*j*_*d,St*_ = (*j* – *j*_*d,St*_)/*j*_*d,St*_ *·*100%, where *j* are the experimental values (reflect the influence of several key components), *j*_*d,St*_ is the calculation on expression (6) (*V*_0_ = 250 μl; *T*_w_ = 75 °C): curve 1 is for the distillate; curve 2 - CaCl_2_ (the initial mass concentration of salt *C*_01_ = 0.2); and curve 3 - LiBr (*C*_01_ = 0.2).

According to 1, the flux *j* always decreases with increasing time, and the rate of this fall decreases with time. The evaporation rate is proportional to the diffusion coefficient *j* $\sim \sqrt{D}$. With time, *j* decreases, and the heat balance changes that leads to an increase in the temperature of the solution. With the temperature increase the diffusion in the liquid increases as well, which partially strengthens the flow *j*, but this effect is much smaller than the effect of Δ*C*. Another important feature of non-isothermal evaporation is the change of evaporation heat *r*, not included in the expression 1 but present in the boundary conditions. The evaporation heat *q* = *rj* is present in the equation for heat balance. At a slow quasi-stationary evaporation, the heat flux from the wall to the liquid is equal to the sum of heat fluxes, used for cooling liquid from evaporation and for cooling due to gas free convection. Usually, the free gas convection is neglected; then, the thermal conductivity of gas may be also neglected, as it is much lower than the thermal conductivity of the solution. In this case, the heat of evaporation is used for cooling the fluid. Below it will be shown that the neglect of free convection is impossible, and it not only significantly changes the heat balance but substantially affects the mechanism of vapor transport. The heat of evaporation increases over time as its value is proportional to the concentration of salt. The increase in *r* also changes the rate of evaporation, which is important to consider when modeling. Figure 2(a) demonstrates experimental and calculated data for the evaporation rate of drops of water and aqueous salt solutions. The experimental data correspond to the points 1–3, and curves 4–6 are obtained by simulation (points 1, 4 – for water; curves 2, 5 – for the aqueous salt solution of LiBr and curves 3, 6 – for the aqueous salt solution of CaCl_2_). The evaporation rate for a water droplet *j* = Δ*m*/Δ*t* slightly increases with time, as with a significant reduction of the droplet height the temperature *T*_*s*_ increases by several degrees. Despite the growth of *T*_*s*_ for solutions of salts, *j* decreases over time. In contrast to expression 1, *j* does not decrease at short times, but first increases and then for some time remains constant. This fact is explained by initial droplet heating (the initial liquid temperature is equal to 21 °C). The temperature of the solution increases with time by 9–11 °C (from *t* = 100 s to 700 s), and the difference Δ*T*_*w*_ = *T*_*w*_ − *T*_*s*_ (*T*_*w*_ is the wall temperature under the drop) drops to 4–6 degrees before crystallization. The difference Δ*T*_*w*_ at initial times is equal to 13–15 degrees.

In Fig. 2(a), regions 1 and 2 are separated by a vertical dotted line. For region 1, the evaporation rate is higher for the aqueous salt solution of LiBr, and for region 2, on the contrary, the aqueous salt solution of CaCl_2_ has higher values of *j*. It is generally accepted that the highest absorption efficiency of the heat pump corresponds to the LiBr salt, as this salt has the highest absorption capacity. It is obvious that the highest absorption activity will match the lowest evaporation rate (absorption is the intake of water vapor, and desorption is the removal of water from the solution surface). However, as can be seen from the figure, the desorption (absorption) activity of an aqueous salt solutions depends on the salt concentration. Before the time 320–340 s (concentration for salts corresponds to the values of about 40–45%) the salt solution of LiBr evaporates faster than the salt solution of CaCl_2_. When the same solution approaches the point of crystallization at the time of 600–800 seconds (the work area of absorption heat pumps) the evaporation rate of the LiBr solution is several times lower than for CaCl_2_. The absorption efficiency will be significantly higher for the highly concentrated LiBr solution. Expressions for concentration may be replaced by the ratio of densities. As the vapor at atmospheric pressure is an ideal gas, in accordance with the Mendeleev-Clapeyron equation the value of the vapor density *ρ* is related to partial vapor pressure *p* by the ratio *ρ* = *pM*/*RT*, where *M* is the molar mass of vapor. The equilibrium vapor pressure corresponds to the curves in Fig. 1(b).

The following regimes are implemented depending on the regime of droplet evaporation: constant contact line (radius) (CR), constant contact angle (CA), and a regime with a sliding and jumping contact line. The simplest case of the CA regime is, when evaporation rate corresponds to power 1/3^50^

2 $$dm/dt={k}_{1}{m}^{1/3},$$

where *k*_1_ is the coefficient independent on mass. From (2), we can derive the expression for a change in the droplet mass with time

3 $${(m/{m}_{0})}^{2/3}=1-{k}_{2}t;\,m/{m}_{0}={(1-{k}_{2}t)}^{3/2},$$

There is no simple solution under the CR regime, and approximations for the evaporation rate (4), taking into account the contact angle *θ*, are often used^49^.

4 $${j}_{d}=\frac{dm}{dt}=-\pi rD{\rm{\Delta }}\rho f(\theta )=-\frac{\pi rDM({p}_{s}-{p}_{o})}{R{T}_{s}}f(\theta ),f(\theta )=1.3+0.27{\theta }^{2}$$

where *r* is the droplet radius, *M* is the molar mass, *p*_*s*_ is the equilibrium vapor pressure at the interface, ∆*ρ* = *ρ*_*s*_ − *ρ*_*∞*_ *, ρ*_*s*_ is the equilibrium vapor density near the interface, *ρ*_*∞*_ is the vapor density of air, *D* is the diffusion coefficient, and *f*(*θ*) is the function of the contact angle *θ*, taken according to the approximation in ref.^49^. The exact theoretical value for zero contact angle is *f*(*θ*) = *f*(0) = 4/*π*. For low contact angles *θ* < 30°, the function is simplified and is approximately equal to 4/*π*, and the expression for *j*_*d*_ takes the form (5) (the subscript *d* takes into account only the vapor diffusion).

5 $${j}_{d}=\frac{dm}{dt}=-4RD{\rm{\Delta }}\rho $$

Large droplets (the droplets of large volume *V*_0_ = 250 µl were used in our experiments) correspond well to (5) even for large angles *θ* ^13^. The calculation of the evaporation rate taking into account the diffusion and the Stefan flow corresponds to (6)^42,43^, and the calculated curves (4–6) are shown in Fig. 2(a) (the subscript *d,St* takes into account the diffusion of vapor and the Stefan flow)

6 $${J}_{d,St}=\frac{dm}{dt}=-4RD({\rho }_{v}+{\rho }_{a}){ln}(1+{B}_{M}),$$

where *B*_*M*_ is the Spalding mass number, ${B}_{M}=\frac{{Y}_{vs-}{Y}_{v\infty }}{1-{Y}_{vs}}$, *Y*_*vs*_ is the mass fraction of vapour/air at the droplet interface, ${Y}_{vs}={(1+(\frac{p}{{p}_{sat}}-1)\frac{{M}_{a}}{{M}_{v}})}^{-1}$, *M* is the molar mass, and *p* is the ambient pressure.

In the evaporation of a sessile droplet at room temperature and in the one at low heat flux when convection is absent, the expression (6) describes the experimental data quite well. Experiments were carried out for the evaporation of droplets with a diameter of 3mm on the wall with a temperature of 30 °C. For this case the experimental data on evaporation rate are described by the expression (6) with a relative error within 6%.

For the case of evaporation of a drop of an aqueous salt solution, *ρ*_*s*_ decreases continuously with time. However, in this case one may use expressions (5, 6) as well. The curve *dm*/*dt* for an aqueous salt solution can be divided into time intervals Δ*t*, and for each interval Δ*ρ*_*s*_ may be considered quasi-constant. As Δ*ρ*_*s*_ is taken from experimental data, the calculated curve will properly reflect the nonstationary character of evaporation. Curve 4 is obtained for water, and curves 5 and 6 are obtained for salt solutions of LiBr and CaCl_2_. As can be seen from the figure, the neglect of convection in expressions (4–6) leads to a multiple understating of the evaporation rate. To calculate *j*_*d,St*_ on (6) it is necessary to know the value of vapor density for the interface at each point in time. The vapor density is determined by the values of partial vapor pressure from the graphs in Fig. 1(b) and given the current concentration of vapor. In order to evaluate how the role of convection changes during the growth of salt concentration, the data in Fig. 2(b) are processed in a dimensionless form Δ_1_ = Δ*j*/*j*_*d,St*_ = (*j* − *j*_*d,St*_)/*j*_*d,St*_ *·*100%, where *j* is the experimental values (reflect the influence of all key components), and *j*_*d,St*_ is the calculation on expression (4), reflecting the influence of diffusion and Stefan flow. As can be seen from Fig. 2(b), for water droplets the excess of free convection over the calculation on (6) is about 140%. For a drop of aqueous salt solution, the dependence is much more complicated than for water. There is a clearly pronounced extremum and three characteristic regions. The region 1 is characterized by a quasi-constant value Δ_1_ = Δ*j*/*j*_*d,St*_, as there is little change in the salt concentration. For region 2, the evaporation rate drops sharply (this decline can be seen from Fig. 2(a)), and the percentage part of convection, on the contrary, increases. The region 3 is characterized by a reduced effect of convection. The reason for the decrease in the influence of convection for the region 3 may be associated with different relationship for the thicknesses of boundary layers (the ratio of the thickness of the dynamic boundary layer *δ*_*a*_ for air to the thickness of the gas-vapor layer above the drop *δ*_*v*_)^14^. Under intense evaporation (stage 1), the ratio of the layers over the drop is markedly lower than for stage 3, i.e., apparently (*δ*_*a*_/*δ*_*v*_)_1_ << (*δ*_*a*_/*δ*_*v*_)_3_. The closer this ratio to 1, the more pronounced the effect of the natural convection.

**Figure 3:**
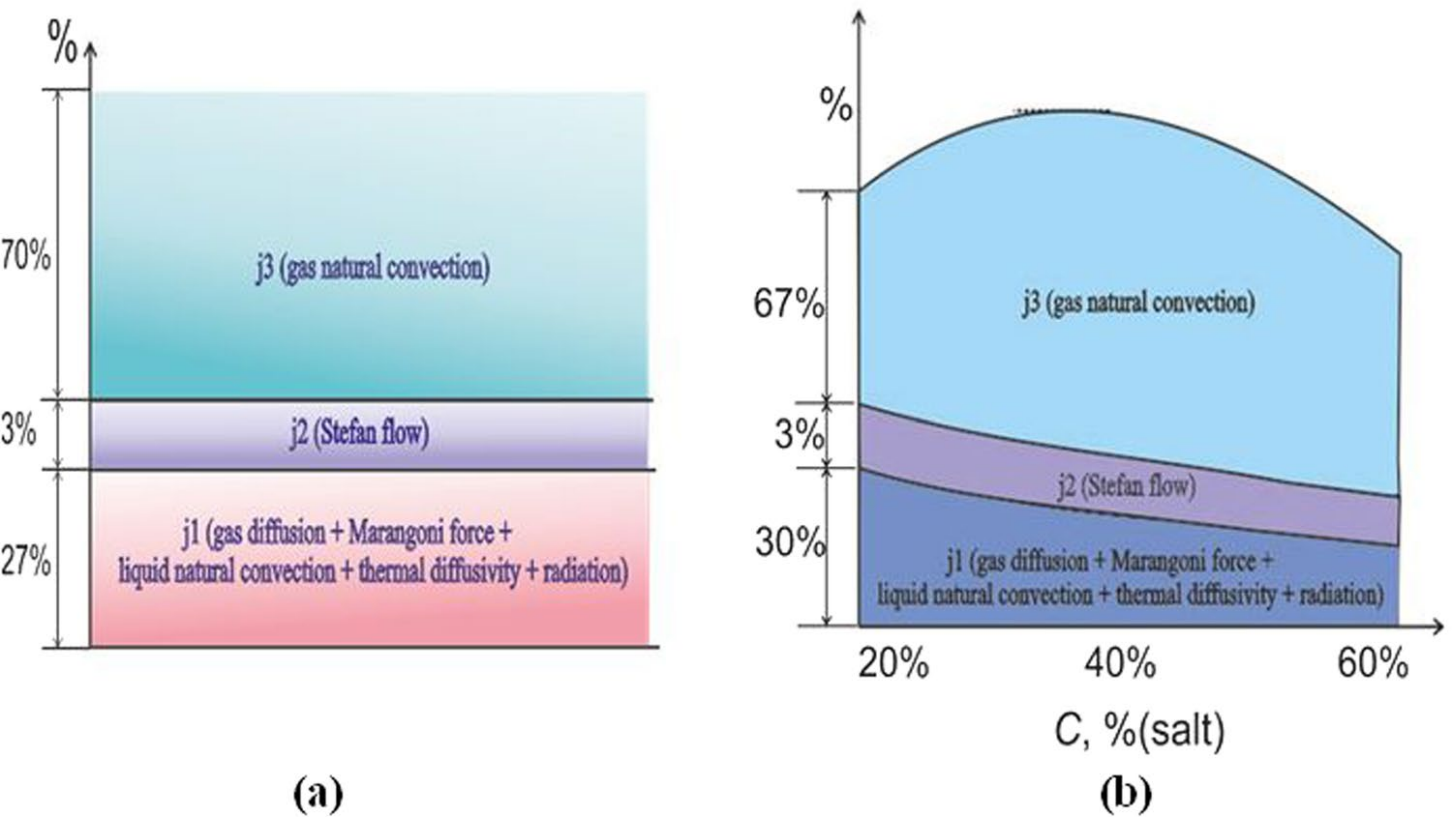
(**a**) The influence (in percent) of various key factors on the evaporation of water drops (*V*_0_ = 250 μl; *T*_w_ = 75 °C); (**b)** The influence (in percent) of various key factors on the evaporation of a drop of aqueous salt solution of LiBr (the initial mass concentration of salt *C*_01_ = 0.2, and the initial drop volume *V*_0_ = 250 μl; *T*_w_ = 75 °C).

**Figure 4:**
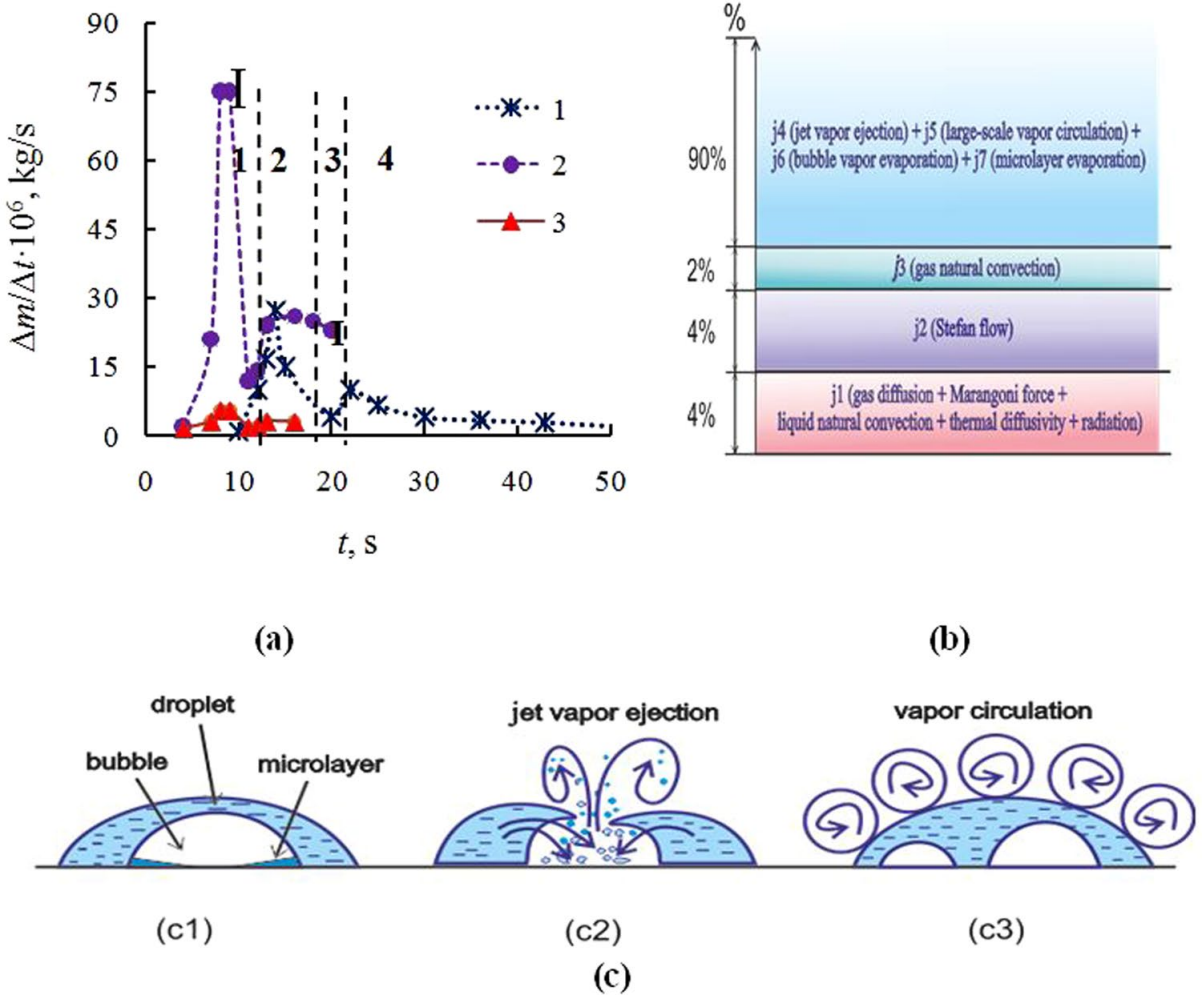
(**a**) Change in the rate of evaporation of salt solution of LiBr and the rate of water evaporation depending on time (1–2 – experiment, 3 - calculation on (6), *V*_0_ = 250 μl; *T*_w_ = 115 °C): 1 – LiBr (*C*_01_ = 0.2); 2–3 – water; (**b)** Schematic diagram on the influence (in percent) of various key factors on water evaporation in the presence of intensive nucleate boiling in the droplet (*V*_0_ = 250 μl; *T*_w_ = 115 °C); (**c**) Evaporation with nucleate boiling in the drop.

Figure 3(a,b) presents schematic diagrams of the effect of key factors on evaporation. Figure 3(a) describes the evaporation of a water drop, and Fig. 3(b) presents evaporation of a drop of aqueous salt solution. As can be seen from Fig. 3, the effect of natural convection for the distillate drop and the drop of the aqueous salt solution varies both qualitatively and quantitatively. In Figs 2(b), 3(a,b) and 4(b), percentage was calculated in different ways. In Fig. 2(b), the difference ∆_1_ = ((*j* − *j*_*d,St*_)/*j*_*d,St*_)·100% was determined. In Figs 3(a,b) and 4(b), the total effect of all factors corresponds to the experimental evaporation rate *j*, treated as 100%. The sum *j*_1_ + *j*_2_ + *j*_3_ corresponds to 100%.The evaporation rate *j* may be considered as a sum of several key factors *j* = *j*_1_ + *j*_2_ + *j*_3_ (Fig. 3), where *j*_1_ is the gas diffusion + Marangony force + liquid natural convection + thermal diffusivity + radiation, *j*_2_ is the Stefan flow, and *j*_3_ is the gas natural convection. In the present work we do not simulate thermo-gravitational convection in the droplet, Marangoni convection, radiation, liquid evaporation heat, heat of dilution, etc. However, these factors affect the interfacial surface temperature of the droplet *T*_*s*_, which was continuously measured by the thermal imager.

Thus, the experimentally measured values of *T*_*s*_ correctly reflect the total effect of all mentioned factors of *j*_1_ in Figs 3(a,b) and 4(b). At that, these factors are not separated or treated individually, and can only be judged on their total impact, which is substantially less than the convection effect. Since the rate of evaporation of water droplet is quasi-constant over time (Fig. 2(a)), the total contribution of various factors (*j*_1_, *j*_2_, *j*_3_) will also be quasi-constant (Fig. 3(a)) (the effect of convection does not change, since *R* = const, and *T*_*s*_ is also quasi-constant for the most evaporation time). The change in the total effect of components of *j*_1_ would also change the rate of drop evaporation. When reducing the height of the droplet, the convection in the liquid decreases^1,2^. Thus, the role of conductive heat transfer increases. As a result of interaction of these two factors, the interfacial surface temperature only slightly increases with time, which leads to a slight increase in the rate of evaporation for water drops. For a drop of aqueous solution of salt (Fig. 3(b)), the change in the salt concentration eventually leads to a change in each component *j*_1_, *j*_2_, *j*_3_. Experimentally determined values *ρ*_*s*_ = *f*(*T*_*s*_) are included in expressions (4–6) and correctly reflect the factors *j*_1_ and *j*_2_. Convection of *j*_3_ is determined as a difference between the experimental and calculated values.

The question of the quantitative effect of gas convection is relevant and is the subject of studies for each individual problem. We introduce the ratio of characteristic rates *a* = *U*_*c*_/*U*_*d*_, where *U*_*c*_ is the characteristic velocity of free gas convection, *U*_*c*_~$\sqrt{\frac{2gl{\rm{\Delta }}\rho }{{\rho }_{mix}}}$, where *l* is the characteristic length, *ρ*_*mix*_ is the density of gas mixture, *U*_*d*_ is the characteristic velocity of vapor diffusion, *U*_*d*_ = *j*_*d*_/(*ρ*_*v*_ *F*), *ρ*_*v*_ is the vapor density, and *F* is the free surface area of the droplet. Since *j*_*d*_ is a complex function of the droplet size, *Ma* numbers, convection in the liquid, wettability, etc., the parameter *a* also depends on many key factors. For very small droplets (*R*_0_ < 1 mm) and for low values of *Gr* numbers for liquid and gas medium, the parameter *a* << 1. In this case we can consider only the diffusion transfer in accordance with expressions (4–6). If *a* >> 1 (high *Gr* numbers) it is necessary to consider the effects of gas convection on the drop evaporation rate. A significant effect of convection in the liquid begins already for drops with *R*_0_ > 2 mm and at Δ*T*_*s*_ = 10 °C^14^. The characteristic rate of the diffusion transfer in a gas may also be determined by *U*_*d*_ = *D*/*δ*_*v*_, where *δ*_*v*_ is the effective thickness of the diffusion vapor layer above the drop surface. It is known that *δ*_*v*_ is approximately equal to the diameter of the drop. Evaluation of the diffusion rate on the experimental values of the evaporation rate and the diffusion coefficient in accordance with data in ref.^38^ show close values of the order of 5 mm/s. For the implementation of convection there is also a need in the condition *Ra* > *Ra*_*cr*_ (where *Ra*_*cr*_ = 1000–2000 is the critical Rayleigh number)^51^.

## The evaporation rate with nucleate boiling in the drop

It is well known that for the attached contact line, for isothermal case and for small contact angle of the drop the derivative of the drop mass on time is constant, and at that, the slope of the straight line depends on temperature, droplet size, and gas diffusion. This behavior is typical of drop evaporation without boiling. At intensive nucleate boiling in the droplet, there are several characteristic regimes of evaporation: 1) after initial heating of the fluid begins nucleate boiling in the droplet. During the first 3–5 s the radius of the droplet increases significantly. 2) The following mode corresponds to the quasi-attached contact line when a change in the droplet radius is less than 20%. This regime is specific for 80–90% of the total evaporation time. 3) The third mode for the sliding contact line (sharp decrease of the radius) is realized when the residual mass of the droplet becomes less than 10%. Thus, it is justified to use the expression (6) for mode 2. The average rate of water drop evaporation was also measured and was determined for each point in time, taking into account changes in the current radius of the droplet (measuring with video camera). The difference of the average evaporation rate *j* from *j* for the second mode was in the range of 4–6%. For the salt solution after initial heating and expansion of the drop (within the first 5–7 s), the drop radius remained constant during the whole measuring time. Changes in the evaporation rate of LiBr aqueous solution and the water evaporation rate are presented in Fig. 4(a). Curve 1 represents experimental data for the aqueous solution of LiBr, curve 2 – for the water droplet, and curve 3 is obtained by calculation on expression (6). It is seen that for water drops (curve 2), the rate of evaporation for stage 2 is almost three times higher than the evaporation rate for the fourth stage for the salt solution. Four characteristic time intervals for LiBr aqueous solution are shown in Fig. 4(a). An intense nucleate boiling for stage 1 leads to a dramatic expansion of the drop. As a result of nucleate boiling, the contact line of the droplet loses its stability. The condition of attached contact line is violated, when the inertial forces are significantly higher than the potential barrier of the contact line. As a result of boiling, the droplet diameter increases from 7–9 mm to 16–19 mm, and the area of drops increases more than twice. When the liquid washes over the hot dry surface of the wall, then the evaporation rate sharply increases. The metal wall for 5–7 seconds fails to compensate cooling due to thermal inertia of the metal and because of a sharp increase in the evaporation rate. The temperature under the wall depends on the diameter of the droplet^8,37^. This scenario corresponds to the time interval 2. After a few seconds, the wall temperature under the droplet increases, and the evaporation rate also increases for section 3. An analogous behavior of the wall temperature under the droplet is observed for the case of drops, falling on the wall^52^. A further increase in salt concentration for interval 4 leads to a significant decrease in the evaporation rate. The evaporation character for the water drop (curve 2) is qualitatively different from the aqueous salt solution (curve 1) only for the time interval 4. For the final time stage, the evaporation rate of the water drop is quasi-constant.

It is important to note the fundamental difference between the evaporation of drops of a salt solution and water drops in the presence of intense bubble boiling. In case of water, the bubble boiling continues for the most evaporation time. However in a salt solution, boiling lasts for a short time interval of 6–8 s, which is approximately 10–20% of the time from the beginning of evaporation to the beginning of crystallization. In the process of boiling, the salt concentration inside the drops abruptly increases, and as a result, the boiling quickly stops. Discontinuation of boiling is associated with a shift of balance. Higher salt concentrations require more overheating for the emergence and growth of vapor bubbles. In the absence of boiling, the evaporation rate in a droplet of an aqueous salt solution is dramatically reduced.

It is obvious that such a drastic change in boundary conditions (changing wall temperature under the drop) greatly affects the calculation accuracy, if the nonstationary nature of evaporation is neglected, and the non-stationary problem is solved for the temperature distribution in the solid wall.

Figure 4(b) presents the role of the key factors (in percent) responsible for evaporation in the presence of intensive nucleate boiling in the droplet. This figure presents non-isothermal evaporation for a water drop. However, for evaporation of a drop of an aqueous salt solution, a substantially prevailing role of parameters *j*_4_-*j*_7_ is also fair. Flows *j*_1_ also indirectly take into account the Marangoni force, natural convection of liquid, thermal diffusivity and radiation, as their superposition impacts the interfacial drop temperature, which is measured by thermal imager.

As previously indicated, only the total effect of several factors is indicated in the graph for *j*_1_ and *j*_4,_ and their individual influence is not considered.

Figure 4(c) schematically represents the mechanism of generation and release of vapor from a drop at nucleate boiling. We have previously shown that modes of vaporization in a drop with nucleate boiling are fundamentally different from the evaporation in a large volume (pool boiling)^9,53^. The evaporation mechanism consists of three main parts: (c1) vapor generation by the near-wall microlayer (microlayer evaporation) and by the bubble surface; (c2) the release of vapor due to the bubble collapse, and the generation of vapor due to the fact that the liquid washes the dry hot surface of the wall (jet vapor ejection); (c3) over the drop surface there is large-scale circulation of vapor *D*_*ω*_, which significantly exceeds the molecular vapor-air diffusion *D* (*D*_*ω*_ >> *D*). It is obvious that the rates of vapor generation and removal depend on the number of bubbles in the droplet *N* and the frequency of bubble generation *ν*_1_, being functions of drop radius (*r*) and drop height (*h*), *j*~*N*(*r*, *h*)^n1^ *ν*_1_(*r*, *h*)^n2^ that fundamentally differs the nucleate boiling in drops from the pool boiling, which is independent from the layer height^53^. In addition, the mechanism of vapor transport over the liquid layer is not considered for pool boiling. The resistance of vapor-gas layer over the drop and the mechanism of vapor transport play a very important role in the case of drops.

It is interesting to note that the Stefan flow at nucleate boiling in a droplet is comparable with *j*_1_ (Fig. 4(b)), in contrast to Fig. 3(a,b), when Stefan flow may be neglected. However, the sum of flows *j*_1_-*j*_3_ (Fig. 4(b)) is negligible compared to *j*_4_-*j*_7_. From the above the important conclusions may be drawn: convection in the liquid during the drop nucleate boiling does not play a significant role and vice versa, gas convection due to vapor release (*j*_4_) and its circulation (*j*_5_) should be taken into account when simulating the droplet evaporation in the presence of intensive nucleate boiling.

## Conclusion

The combined effect of gas convection, the Stefan flow and vapor diffusion in the presence of high heat flux have been studied. The evaporation character of droplets of aqueous salt solutions is fundamentally different from that in volatile single-component liquids. The study of the influence of concentrations of solution components on the qualitative and quantitative behavior of a droplet at high wall temperatures is a major scientific challenge and is of interest to a wide range of modern technologies.

It is known that the higher absorption efficiency in an absorber of heat pump corresponds to a highly concentrated aqueous salt solution of LiBr. The highest absorption efficiency of an aqueous salt solution corresponds to the lowest evaporation rate. However, the absorption and the desorption of aqueous solutions depend on the salt concentration. Up to salt concentration of about 40%, the aqueous salt solution of LiBr evaporates faster than that of CaCl_2_. And when the solution approaches the crystallization point (the working area of the absorption heat pump) the evaporation rate of the LiBr solution is several times lower than that of CaCl_2_. The absorption efficiency is maximal for highly concentrated LiBr solution.

At non-isothermal evaporation of salt solutions there are a pronounced extremum and three characteristic time intervals. Interval 1 is characterized by a quasi-constant ratio of gas convection to vapor diffusion, as there is little change in the salt concentration. For interval 2, the drop evaporation rate decreases sharply, and the role of gas convection, on the contrary, grows. Time interval 3 is characterized by a decrease in the role of convection. The reason for a significant drop of the convection influence for the region 3 may be associated with thicknesses of gas boundary layers (the ratio of thickness of the dynamic boundary layer *δ*_*a*_ for air to thickness of the vapor layer above the drop *δ*_*v*_).

The effect of the key factors in droplet evaporation is qualitatively and quantitatively different in cases of nucleate boiling and without it. Despite that in the nucleate boiling, the Stephan flow is comparable with the diffusive gas flux, and a significant part of droplet evaporation is related to the mechanisms of vapor generation and transport by vapor bubbles. At that, the vapor transport mechanism in a drop is qualitatively different from that at pool boiling.

At intensive nucleate boiling in a drop of water or an aqueous salt solution, the stability of the drop contact line is violated, and the wall temperature under the drop significantly changes. As a result, there are four evaporation modes when the evaporation rate significantly changes. The last fourth regime of the water drop evaporation is characterized by a quasi-constant evaporation rate. For a drop of salt solution, the evaporation rate in the fourth mode decreases many times, and as a result, the impact of free gas convection significantly increases. Thus, at simulating high temperature evaporation of drops, it is necessary to take into account changes in boundary conditions with time, i.e. the change in both the wall temperature under the droplet (it is necessary to solve the conjugated problem of heat transfer between a solid wall and a liquid droplet), and in the intensity of vapor-gas natural convection. These boundary conditions change with the growth of salt concentration over time.
